# Supplementary material for: Reproducible and clinically translatable deep neural networks for cervical screening
Source: Sci Rep. 2023 Dec 8;13:21772. doi: 10.1038/s41598-023-48721-1 (PMC10709439; doi:10.1038/s41598-023-48721-1)
Supplement: Supplementary file 1 — Supplementary Information. [file 41598_2023_48721_MOESM1_ESM.pdf]

SUPPLEMENTARY INFORMATION

SUPPLEMENT SECTION 1: SUPPLEMENTARY METHODS

(A) INDIVIDUAL DATASET DESCRIPTIONS

(i) Natural History Study (NHS)

The Natural History Study (NHS) is a population-based prospective study carried out in Guanacaste Costa Rica between 1993 and 2000 [1]. This cohort enrolled women followed in either an active cohort with visits every 6-12 months or a passive cohort screened once during follow-up between 5-7 years after enrollment. Screening visits included collection of specimens for cytology, human papillomavirus (HPV) testing, and digital images, while histology was collected among women with abnormal colposcopic evaluation. Cytology was assessed via both conventional and liquid-based methods as well as a first-generation automated approach. HPV testing by MY09/MY11 polymerase chain reaction (PCR) consensus primers was performed on samples collected by Dacron swabs, however, these results were not used for colposcopy referral during the study. Two cervical images per visit were collected at each screening visit using a Cervigram cerviscope, which were later digitized and compressed for storage [2].

(ii) ASCUS/LSIL Triage Study for Cervical Cancer (ALTS)

The ASCUS/LSIL Triage Study for Cervical Cancer (ALTS) is a multi-center randomized trial of US women conducted between 1996 and 2000. This study enrolled women attending colposcopy clinics with referral cytology of either atypical squamous cells of undetermined significance (ASCUS) or low-grade squamous intraepithelial lesion (LSIL). Women were followed for 2 years with screening visits every 6 months. Screening visit specimen collection included two cervical specimens, one for liquid-based cytology and one for HPV testing, as well as cervical images. Referral to colposcopy and histologic sampling varied by study visit, including enrollment referral following the referral cytology result as well as the randomized HPV result, referral from follow-up visit due to high-grade squamous intraepithelial lesion (HSIL) cytology, and exit colposcopy for all women. Type-specific HPV results were not used for patient management [3]. Cytologic diagnosis were based on ThinPrep slides created from

cytobrush collected exfoliated cells eluted into PreservCyt-media specimens, with both clinical and quality control (QC) evaluations performed. HPV typing was performed by PCR on specimens collected in PreservCyt. A cerviscope was used to collect two images per screening visit and were later converted to a digital format in the same process used for NHS images.

(iii) Costa Rica Vaccine Trial (CVT)

The CVT study is a double-blind, controlled, randomized, phase III study of the efficacy of an HPV16/18 virus-like particle (VLP) vaccine in the prevention of advanced cervical intraepithelial neoplasia (cervical intraepithelial neoplasia (CIN) 2, CIN3, adenocarcinoma in situ (AIS) and invasive cervical cancer) associated with HPV 16 or HPV 18 cervical infection in healthy young adult women in Costa Rica, Guanacaste, and parts of the Puntarenas provinces [4]. Women were randomized to either the HPV16/18 or control group and followed up for 4 years as part of this study. Images were collected from women who were only referred for colposcopic evaluation, who remained at colposcopy until they had two consecutive results within normal limits. Images were acquired using a Nikon digital single-lens reflex (DSLR) camera with a beam splitter of colposcopy imaging and were subsequently collected using a boundary marking tool.

(iv) Biopsy study (Biop):

The Biopsy Study (Biop) was a population-based study of women referred to colposcopy for abnormal cervical cancer screening results conducted at the University of Oklahoma Health Sciences Center (OUHSC) from February 2009 to August 2011, designed with the goal of utilizing biopsies to improve detection of cervical precancer. HPV testing was conducted via the LINEAR ARRAY® multiplexed PCR-based assay. Histologic interpretation of biopsy and LEEP specimens was conducted using CIN terminologies. All women enrolled in the study had a colposcopy performed and at least one biopsy. Images were acquired using a Nikon DSLR camera with a beam splitter of colposcopy imaging and were subsequently annotated and collected using the boundary marking tool [5].

(v) Biopsy Study – Europe (D Biop)

Fifth, we used data and images from a European study (D Biop) designed to investigate high-risk HPV genotypes in women with histologic CIN2/3 referred on the basis of abnormal cytology. HPV typing was done on cytology and CIN2/3 biopsies. If the whole-tissue section of the biopsy was positive for multiple high-risk HPV types, LCM-PCR was performed. Images were acquired using a DSLR camera [6].

SUPPLEMENT SECTION 2: SUPPLEMENTARY TABLES AND FIGURES

Histology	Cytology	HPV	Study				
			NHS	ALTS	CVT	Biop	D Biop
Cancer			Cancer	Cancer	Cancer	Cancer	Cancer
CIN3/AIS			Precancer	Precancer	Precancer	Precancer	Precancer
CIN2		Onco+	Precancer	Precancer	Precancer	Precancer	Precancer
		Onco-	Gray High	Gray High	Gray High	Gray High	Gray High
		Missing	Gray High	Gray High		Gray High	Gray High
CIN1		Onco+	Gray Middle				
Normal or no histology	Multiple HSIL	HPV16+	Precancer				
		Onco+, not HPV16	Gray High				
	HSIL	Onco+	Gray Middle	Gray High	Gray High	Gray High	Gray High
		Onco-	Gray Low	Gray Low	Gray Low	Gray Low	Gray Low
		Missing	Gray Low	Gray High	Gray High		Gray High
	ASCUS/LSIL	Onco+	Gray Middle	Gray Middle	Gray Middle	Gray Middle	Gray Middle
	LSIL	Onco-	Gray Low	Gray Low	Gray Low	Gray Low	Gray Low
	ASCUS	Onco-	Normal	Normal	Normal	Normal	Normal
		Missing	Normal	Gray Low	Gray Low		Gray Low
	NILM	Onco+	Gray Low	Gray Low	Gray Low	Gray Low	Gray Low
		Onco-	Normal	Normal	Normal	Normal	Normal
		Missing		Normal	Normal	Normal	Normal
	Missing	Onco+					Gray Low
		Onco-					Normal

Supplementary Table 1. Detailed breakdown of ground truth definitions by study.

Supplementary Table 2: Detailed breakdown of full 5-study dataset by set (train, validation, test 1 or test 2), study and ground truth																
STUDY	GROUND TRUTH CATEGORIES no. (%)												GRAND TOTAL BY STUDY (n_i=17013, n_w=9462) no. (%)			
	Normal (n_i=11630, n_w=6092)				Gray Zone (n_i=3586, n_w=2314)				Precancer+ (n_i=1797, n_w=1056)							
	# images		*# women*		*# images*		*# women*		*# images*		*# women*		*# images*		*# women*	
**Train Set**																
NHS	5407	(77.4%)	2711	(74.2%)	330	(15.3%)	165	(11.9%)	206	(19.0%)	104	(16.4%)	5943	(58.1%)	2980	(52.4%)
ALTS	1129	(16.2%)	566	(15.5%)	853	(39.6%)	430	(30.9%)	434	(40.1%)	218	(34.3%)	2416	(23.6%)	1214	(21.4%)
CVT	253	(3.6%)	253	(6.9%)	336	(15.6%)	335	(24.1%)	121	(11.2%)	119	(18.7%)	710	(6.9%)	707	(12.4%)
Biop	93	(1.3%)	40	(1.1%)	192	(8.9%)	88	(6.3%)	164	(15.2%)	79	(12.4%)	449	(4.4%)	207	(3.6%)
D Biop	105	(1.5%)	85	(2.3%)	444	(20.6%)	374	(26.9%)	157	(14.5%)	116	(18.2%)	706	(6.9%)	575	(10.1%)
TOTAL	6987	(100.0%)	3655	(100.0%)	2155	(100.0%)	1392	(100.0%)	1082	(100.0%)	636	(100.0%)	10224	(100.0%)	5683	(100.0%)
(a)	68.3%		64.3%		21.1%		24.5%		10.6%		11.2%		100.0%		100.0%	
(b)													60.1%		60.1%	
**Validation Set**																
NHS	903	(77.6%)	452	(73.6%)	55	(15.1%)	28	(12.3%)	34	(19.2%)	17	(16.7%)	992	(58.2%)	497	(52.6%)
ALTS	187	(16.1%)	94	(15.3%)	142	(39.0%)	71	(31.1%)	72	(40.7%)	36	(35.3%)	401	(23.5%)	201	(21.3%)
CVT	48	(4.1%)	48	(7.8%)	53	(14.6%)	53	(23.2%)	17	(9.6%)	17	(16.7%)	118	(6.9%)	118	(12.5%)
Biop	10	(0.9%)	6	(1.0%)	35	(9.6%)	14	(6.1%)	29	(16.4%)	13	(12.7%)	74	(4.3%)	33	(3.5%)
D Biop	15	(1.3%)	14	(2.3%)	79	(21.7%)	62	(27.2%)	25	(14.1%)	19	(18.6%)	119	(7.0%)	95	(10.1%)
TOTAL	1163	(100.0%)	614	(100.0%)	364	(100.0%)	228	(100.0%)	177	(100.0%)	102	(100.0%)	1704	(100.0%)	944	(100.0%)
(a)	68.3%		65.0%		21.4%		24.2%		10.4%		10.8%		100.0%		100.0%	
(b)													10.0%		10.0%	
**Test Set 1**																
NHS	1798	(77.3%)	903	(74.1%)	108	(15.3%)	55	(11.9%)	70	(19.1%)	35	(16.2%)	1976	(58.1%)	993	(52.3%)
ALTS	376	(16.2%)	189	(15.5%)	285	(40.3%)	143	(31.0%)	146	(39.8%)	73	(33.8%)	807	(23.7%)	405	(21.3%)
CVT	86	(3.7%)	86	(7.1%)	110	(15.6%)	110	(23.8%)	42	(11.4%)	42	(19.4%)	238	(7.0%)	238	(12.5%)
Biop	30	(1.3%)	13	(1.1%)	60	(8.5%)	29	(6.3%)	55	(15.0%)	27	(12.5%)	145	(4.3%)	69	(3.6%)
D Biop	35	(1.5%)	28	(2.3%)	144	(20.4%)	125	(27.1%)	54	(14.7%)	39	(18.1%)	233	(6.9%)	192	(10.1%)
TOTAL	2325	(100.0%)	1219	(100.0%)	707	(100.0%)	462	(100.0%)	367	(100.0%)	216	(100.0%)	3399	(100.0%)	1897	(100.0%)
(a)	68.4%		64.3%		20.8%		24.4%		10.8%		11.4%		100.0%		100.0%	
(b)													20.0%		20.0%	
**Test Set 2**																
NHS	902	(78.1%)	452	(74.8%)	54	(15.0%)	27	(11.6%)	34	(19.9%)	17	(16.7%)	990	(58.7%)	496	(52.9%)
ALTS	187	(16.2%)	94	(15.6%)	144	(40.0%)	72	(31.0%)	72	(42.1%)	36	(35.3%)	403	(23.9%)	202	(21.5%)
CVT	37	(3.2%)	37	(6.1%)	56	(15.6%)	56	(24.1%)	17	(9.9%)	17	(16.7%)	110	(6.5%)	110	(11.7%)
Biop	14	(1.2%)	7	(1.2%)	28	(7.8%)	15	(6.5%)	27	(15.8%)	13	(12.7%)	69	(4.1%)	35	(3.7%)
D Biop	15	(1.3%)	14	(2.3%)	78	(21.7%)	62	(26.7%)	21	(12.3%)	19	(18.6%)	114	(6.8%)	95	(10.1%)
TOTAL	1155	(100.0%)	604	(100.0%)	360	(100.0%)	232	(100.0%)	171	(100.0%)	102	(100.0%)	1686	(100.0%)	938	(100.0%)
(a)	68.5%		64.4%		21.4%		24.7%		10.1%		10.9%		100.0%		100.0%	
(b)													9.9%		9.9%	
**GRAND TOTAL BY GROUND TRUTH**																
no. (%)	11630 (68.4%)		6092 (64.4%)		3586 (21.1%)		2314 (24.5%)		1797 (10.6%)		1056 (11.2%)		17013 (100.0%)		9462 (100.0%)	

Supplementary Table 2: Detailed breakdown of full 5-study dataset by set (train, validation, test 1, test 2), study and ground truth. n_i=total # images; n_w=total # women; (a) Ground truth ratios (by images or women) within each set (train/validation/test 1/test 2) = Total # (images or women) in the ground truth category of set ÷ Total # (images or women) in the set; (b) Proportion of total (images or women) in each set (train/validation/test 1/test 2) = Total # (images or women) in the set ÷ Total # (images or women) in the full dataset.

Supplementary Table 3: Detailed breakdown of rebalanced dataset after applying “remove controls” balancing strategy, by set (train, validation, test 1 or test 2), study and ground truth																
**STUDY**	Ground truth categories no. (%)												GRAND TOTAL BY STUDY (n_i=17013, n_w=9462) no. (%)			
	Normal (n_i=11630, n_w=6092)				Gray Zone (n_i=3586, n_w=2314)				Precancer+ (n_i=1797, n_w=1056)							
	# images		*# women*		*# images*		*# women*		*# images*		*# women*		*# images*		*# women*	
**Train Set**																
NHS	1887	(77.6%)	946	(74.4%)	330	(15.3%)	165	(11.9%)	206	(19.0%)	104	(16.4%)	2423	(42.7%)	1215	(36.8%)
ALTS	387	(15.9%)	194	(15.3%)	853	(39.6%)	430	(30.9%)	434	(40.1%)	218	(34.3%)	1674	(29.5%)	842	(25.5%)
CVT	88	(3.6%)	88	(6.9%)	336	(15.6%)	335	(24.1%)	121	(11.2%)	119	(18.7%)	545	(9.6%)	542	(16.4%)
Biop	35	(1.4%)	13	(1.0%)	192	(8.9%)	88	(6.3%)	164	(15.2%)	79	(12.4%)	391	(6.9%)	180	(5.5%)
D Biop	35	(1.4%)	31	(2.4%)	444	(20.6%)	374	(26.9%)	157	(14.5%)	116	(18.2%)	636	(11.2%)	521	(15.8%)
TOTAL	2432	(100.0%)	1272	(100.0%)	2155	(100.0%)	1392	(100.0%)	1082	(100.0%)	636	(100.0%)	5669	(100.0%)	3300	(100.0%)
(a)	42.9%		38.5%		38.0%		42.2%		19.1%		19.3%		100.0%		100.0%	
(b)													33.3%		34.9%	
**Validation Set**																
NHS	291	(76.0%)	146	(71.6%)	55	(15.1%)	28	(12.3%)	34	(19.2%)	17	(16.7%)	380	(41.1%)	191	(35.8%)
ALTS	65	(17.0%)	33	(16.2%)	142	(39.0%)	71	(31.1%)	72	(40.7%)	36	(35.3%)	279	(30.2%)	140	(26.2%)
CVT	19	(5.0%)	19	(9.3%)	53	(14.6%)	53	(23.2%)	17	(9.6%)	17	(16.7%)	89	(9.6%)	89	(16.7%)
Biop	4	(1.0%)	2	(1.0%)	35	(9.6%)	14	(6.1%)	29	(16.4%)	13	(12.7%)	68	(7.4%)	29	(5.4%)
D Biop	4	(1.0%)	4	(2.0%)	79	(21.7%)	62	(27.2%)	25	(14.1%)	19	(18.6%)	108	(11.7%)	85	(15.9%)
TOTAL	383	(100.0%)	204	(100.0%)	364	(100.0%)	228	(100.0%)	177	(100.0%)	102	(100.0%)	924	(100.0%)	534	(100.0%)
(a)	41.5%		38.2%		39.4%		42.7%		19.2%		19.1%		100.0%		100.0%	
(b)													5.4%		5.6%	
**Test Set 1**																
NHS	5930	(77.4%)	2974	(74.1%)	108	(15.3%)	55	(11.9%)	70	(19.1%)	35	(16.2%)	6108	(69.9%)	3064	(65.3%)
ALTS	1240	(16.2%)	622	(15.5%)	285	(40.3%)	143	(31.0%)	146	(39.8%)	73	(33.8%)	1671	(19.1%)	838	(17.9%)
CVT	280	(3.7%)	280	(7.0%)	110	(15.6%)	110	(23.8%)	42	(11.4%)	42	(19.4%)	432	(4.9%)	432	(9.2%)
Biop	94	(1.2%)	44	(1.1%)	60	(8.5%)	29	(6.3%)	55	(15.0%)	27	(12.5%)	209	(2.4%)	100	(2.1%)
D Biop	116	(1.5%)	92	(2.3%)	144	(20.4%)	125	(27.1%)	54	(14.7%)	39	(18.1%)	314	(3.6%)	256	(5.5%)
TOTAL	7660	(100.0%)	4012	(100.0%)	707	(100.0%)	462	(100.0%)	367	(100.0%)	216	(100.0%)	8734	(100.0%)	4690	(100.0%)
(a)	87.7%		85.5%		8.1%		9.9%		4.2%		4.6%		100.0%		100.0%	
(b)													51.3%		49.6%	
**Test Set 2**																
NHS	902	(78.1%)	452	(74.8%)	54	(15.0%)	27	(11.6%)	34	(19.9%)	17	(16.7%)	990	(58.7%)	496	(52.9%)
ALTS	187	(16.2%)	94	(15.6%)	144	(40.0%)	72	(31.0%)	72	(42.1%)	36	(35.3%)	403	(23.9%)	202	(21.5%)
CVT	37	(3.2%)	37	(6.1%)	56	(15.6%)	56	(24.1%)	17	(9.9%)	17	(16.7%)	110	(6.5%)	110	(11.7%)
Biop	14	(1.2%)	7	(1.2%)	28	(7.8%)	15	(6.5%)	27	(15.8%)	13	(12.7%)	69	(4.1%)	35	(3.7%)
D Biop	15	(1.3%)	14	(2.3%)	78	(21.7%)	62	(26.7%)	21	(12.3%)	19	(18.6%)	114	(6.8%)	95	(10.1%)
TOTAL	1155	(100.0%)	604	(100.0%)	360	(100.0%)	232	(100.0%)	171	(100.0%)	102	(100.0%)	1686	(100.0%)	938	(100.0%)
(a)	68.5%		64.4%		21.4%		24.7%		10.1%		10.9%		100.0%		100.0%	
(b)													9.9%		9.9%	
**GRAND TOTAL BY GROUND TRUTH**																
**no. (%)**	11630 (68.4%)		6092 (64.4%)		3586 (21.1%)		2314 (24.5%)		1797 (10.6%)		1056 (11.2%)		17013 (100.0%)		9462 (100.0%)	

Supplementary Table 3: Detailed breakdown of rebalanced dataset after “remove controls” balancing strategy, by set (train, validation, test 1, test 2), study and ground truth. n_i=total # images; n_w=total # women; (a) Ground truth ratios (by images or women) within each set (train/validation/test 1/test 2) = Total # (images or women) in the ground truth category of set ÷ Total # (images or women) in the set; (b) Proportion of total (images or women) in each set (train/validation/test 1/test 2) = Total # (images or women) in the set ÷ Total # (images or women) in the full dataset.

REFERENCES

1. Herrero, R. *et al.* Design and methods of a population-based natural history study of cervical neoplasia in a rural province of Costa Rica: the Guanacaste Project. *Rev. Panam. Salud Publica* **1**, 411–425 (1997).
2. Rodriguez, A. C. *et al.* Longitudinal study of human papillomavirus persistence and cervical intraepithelial neoplasia grade 2/3: critical role of duration of infection. *J. Natl. Cancer Inst.* **102**, 315–324 (2010).
3. ASCUS-LSIL Traige Study (ALTS) Group. A randomized trial on the management of low-grade squamous intraepithelial lesion cytology interpretations. *Am. J. Obstet. Gynecol.* **188**, 1393–1400 (2003).
4. Herrero, R. *et al.* Prevention of persistent human papillomavirus infection by an HPV16/18 vaccine: a community-based randomized clinical trial in Guanacaste, Costa Rica. *Cancer Discov.* **1**, 408–419 (2011).
5. Wentzensen, N. *et al.* Performance of p16/Ki-67 immunostaining to detect cervical cancer precursors in a colposcopy referral population. *Clin. Cancer Res.* **18**, 4154–4162 (2012).
6. Van Der Marel, J. *et al.* Attributing oncogenic human papillomavirus genotypes to high-grade cervical neoplasia: which type causes the lesion? *Am. J. Surg. Pathol.* **39**, 496–504 (2015).
